# Preparing for COVID-19: Household food insecurity and vulnerability to shocks in Nairobi, Kenya

**DOI:** 10.1371/journal.pone.0259139

**Published:** 2021-11-11

**Authors:** Elizabeth Opiyo Onyango, Jonathan Crush, Samuel Owuor

**Affiliations:** 1 Balsillie School of International Affairs, Waterloo, Ontario, Canada; 2 Wilfrid Laurier University, Waterloo, Ontario, Canada; 3 University of the Western Cape, Cape Town, South Africa; 4 University of Nairobi, Nairobi, Kenya; University of Texas at Arlington, UNITED STATES

## Abstract

An understanding of the types of shocks that disrupt and negatively impact urban household food security is of critical importance to develop relevant and targeted food security emergency preparedness policies and responses, a fact magnified by the current COVID-19 pandemic. This gap is addressed by the current study which draws from the Hungry Cities Partnership (HCP) city-wide household food insecurity survey of Nairobi city in Kenya. It uses both descriptive statistics and multilevel modelling using General Linear Mixed Models (GLMM) to examine the relationship between household food security and 16 different shocks experienced in the six months prior to the administration of the survey. The findings showed that only 29% of surveyed households were completely food secure. Of those experiencing some level of food insecurity, more experienced economic (55%) than sociopolitical (16%) and biophysical (10%) shocks. Economic shocks such as food price increases, loss of employment, and reduced income were all associated with increased food insecurity. Coupled with the lack of functioning social safety nets in Nairobi, households experiencing shocks and emergencies experience serious food insecurity and related health effects. In this context, the COVID-19 pandemic is likely to have a major negative economic impact on many vulnerable urban households. As such, there is need for new policies on urban food emergencies with a clear emergency preparedness plan for responding to major economic and other shocks that target the most vulnerable.

## Introduction

Studies on the linkages between emergency shocks and food insecurity have tended to focus on the impacts on household agricultural production in rural areas [1–6]. Less well-researched, especially in the African and Kenyan contexts, are the effects of emergencies and shocks on urban household food security and the health and well-being of urban households [7–9]. The COVID-19 pandemic has provided a major shock to global and local food supply chains, including in Kenya, but it is the over-crowded urban areas that have proved to be the hotspots for virus transmission [10–13]. There is growing evidence that the pandemic constitutes a major disruptive shock to the food security of urban households by reducing the availability of food, increasing food prices, reducing physical accessibility to formal and informal food retail outlets, and hollowing out the labour market with consequent increases in unemployment and reduced household income [10, 12]. This, in turn, has led to a dramatic increase in levels of and vulnerability to food insecurity, especially in cities where households are primarily dependent on food purchase for their daily consumption needs [14–18]. In Kenya, preliminary rapid response research has suggested widespread income shocks and worsening food security and dietary quality as a result of the pandemic and various mitigation and containment measures [19, 20]. At the same time, these shocks have impacted most severely on income-poor households [21].

In urban areas in low-to-middle income countries (LMICs), concerns about food insecurity in times of emergency primarily revolve around risk factors, and the vulnerability of individuals and households [22, 23]. Various types of shocks lead to loss of real income and assets. The (in)ability to secure sufficient income to be able to afford food and other basic needs is often compounded by rising prices of these necessities. While these factors directly impact on food security, other compounding risk factors include overcrowding and unhygienic environments and the absence of functioning social safety nets in most LMIC cities. Using various metrics, recent studies have suggested that having access to stable social grants and remittances can have a positive impact on the nutritional status of urban and rural households [24, 25]. While COVID-19 therefore represents a profound and possibly unprecedented shock to urban household well-being and food security, it is certainly not the first or the last or only major shock to impact on food security at the household and individual level in cities such as Nairobi. Political violence, unemployment, loss of income, climate change, and food price crises are all shocks that have an immediate and negative impact on the availability of food, its accessibility and utilization, as well as disrupting the stability of food supply. The 2007–2008 food price crisis and political violence, for example, were well-documented shocks to urban livelihoods and food security, particularly among the urban poor [26]. Climate change is also affecting domestic food production and, by extension, is leading to increased food imports and rising food prices setting off an inflationary spiral that represents another form of shock to urban households [27].

Food security is commonly defined as a situation “when all people, at all times, have physical, social, and economic access to sufficient, safe, and nutritious food that meets their dietary needs and food preferences for an active and healthy life” and has four main dimensions or pillars–food availability, food accessibility, food utilization and food stability [28]. Previous studies have provided strong evidence of extreme food and nutrition security inequality in Nairobi and a concentration of severely food insecure households in the city’s large informal settlements [9, 29–33]. However, there have been few studies to date on the relationship between a wide range of potential economic, sociopolitical and biophysical shocks and food security impacts at the household level, despite the fact that all four dimensions of food security are susceptible to external emergencies and shocks [1]. By examining aspects of this relationship, this paper provides insights into which kinds of households in Nairobi are food insecure but also whether or not the experience of food insecurity is related to external hazards and household shocks. This, in turn, provides insights into which types of households are most likely to be negatively affected by COVID-19’s impact on food security. In policy terms, the analysis also has lessons for emergency disaster food security planning in the country, contributing to a growing literature on this subject on the pandemic [34–42], and showing which households are most likely to be vulnerable to severe food insecurity and requiring emergency assistance in this and any future emergencies.

In order to develop better emergency food preparedness policies, it is important to identify which types of shocks are most likely to disrupt and impact negatively on household food security and which types of households are most vulnerable to these shocks. This paper therefore draws on data from a pre-pandemic household survey of Nairobi to examine the relationship between household food security and 16 different types of shocks experienced by households in the six months prior to the administration of the survey. The next section of the paper describes the materials and methods used to draw the city-wide sample in the HCP survey and the analytical methods including descriptive statistics and multivariate analysis to ascertain which household characteristics and types of shocks are most likely to be associated with food insecurity.

## Materials and methods

The data for this paper was drawn from the 2017 Hungry Cities Partnership (HCP) urban household food security survey for Nairobi [9]. The survey was a cross-sectional study based on city-wide representative household data. The analytical sample was drawn from a three-stage cluster sampling and probability proportion to size sampling strategy. Nairobi is divided into four administrative districts (sub-counties)–Nairobi West, Nairobi East, Nairobi North and Westlands. These counties are sub-divided into eight administrative divisions: Dagoretti and Kibera (in Nairobi West), Embakasi and Makadara (in Nairobi East), Central, Kasarani and Pumwani (in Nairobi North) and Westlands division (in Westlands). These divisions comprise 49 administrative locations, split into 111 sub-locations (the smallest administrative unit in Kenya.) Twenty-three of the sub-locations were randomly selected and the number of households sampled aimed to be proportional to size in each sub-location. In the sampled sub-locations, systematic random sampling was used to identify participating households where every nth household was recruited and interviewed. The household head or representative was the target interviewee. The surveys were administered using tablets in face-to-face interviews with a team of experienced and trained enumerators from University of Nairobi over a 14-day period. Table 1 shows the selected sub-locations, the number of households in each, and the number surveyed.

**Table 1:** Number of surveyed households by sub-location.

	No. of Households in Sub-Location	No. of Surveyed Households
NAIROBI WEST DISTRICT		
Dagoretti Division		
Kawangware	22,262	192
Kenyatta/Golf Course	5,987	27
Riruta	20,245	94
Kibera Division		
Karen	2,861	21
Lindi	11,551	74
South C	13,759	49
NAIROBI EAST DISTRICT		
Embakasi Division		
Embakasi	19,815	111
Komarock	8,039	46
Umoja	28,097	160
Makadara Division		
Hamza	5,348	65
Makongeni	3,744	43
Hazina	6,445	50
NAIROBI NORTH DISTRICT		
Central Division		
Huruma	23,800	112
Pangani	9,343	58
Ngara East	5,067	30
Kasarani Division		
Zimmerman	10,309	62
Roysambu	9,002	55
Pumwani Division		
Uhuru	6,450	40
Shauri Moyo	5,304	41
Bondeni/Gorofani	1,824	17
WESTLANDS DISTRICT		
Westlands Division		
Highridge	8,075	50
Kileleshwa	4,592	24
Spring Valley	1,378	13

Note: The administrative units are based on 2009 Kenya Population and Housing Census.

The HCP survey instrument contains several questions relevant to this paper, including household and individual demographic characteristics; social and economic profile of the households including employment, income, and expenditure; food security and poverty metrics; household food sourcing challenges and strategies; health status of household members; and household experience of various different types of shock in the months prior to the survey. Sixteen possible shocks were identified and grouped into three broad types: economic shocks, socio-political shocks, and biophysical shocks. Economic shocks included the household going without food due to food price increases, the death or serious illness of a working member of the household, loss of employment or reduced income of a household member, and a reduction or cut-off of remittances. Socio-political shocks included insecurity and violence, theft of money or food, family relocation, and political problems or issues. Biophysical shocks included health risks such as epidemics, fire and, flooding, increases in the cost of water, and lack of storage or refrigeration for food. In the survey, household heads were asked the question: “Did any of the following problems prevent you from having enough food to meet your family’s needs in the past six months? Yes/No.” The health status variable was a self-reported binary (yes/no) response to questions about whether any of the household members had medically diagnosed health issues including non-communicable diseases (diabetes, cancers, heart disease, obesity, high blood pressure and stroke, arthritis, and asthma) and communicable diseases (tuberculosis and diarrhea) at the time of the interview. The individual measures of health were then used to generate binary variables for household health status i.e., whether a household had ‘no health issues’ or ‘some health issues.’ The primary limitation here is that all diseases had to have a medical diagnosis which may have led to undercounting of actual disease prevalence.

The food security status of each household was measured using the Household Food Insecurity Access Prevalence (HFIAP) indicator developed by the Food and Nutrition Technical Assistance (FANTA) project [42]. A score was calculated for each household based on their responses to nine frequency-of-occurrence questions in the four weeks prior to the interview. Scores ranged between 0 and 27 with a score of 0 indicating that the household is food secure, and a maximum score of 27 indicating extreme food insecurity. The answers to the questions were converted into a categorical variable using the FANTA algorithm to generate the HFIAP classification. The HFIAP categorizes households into one of four status levels–food secure, mildly food insecure, moderately food insecure, and severely food insecure. The HFIAP is the preferred measure of household food security as it is adaptable across diverse socio-cultural contexts and captures different dimensions of food insecurity including availability, access, and utility. Furthermore, it is based on a standardized questionnaire and data collection and analysis methodologies that have been widely tested and adopted in the literature [43].

The data were analyzed descriptively using percentages and bar graphs to give a description of the study participants and their households (Table 1). Generalized Linear Mixed Modelling was used in a multivariate analysis to ascertain which household characteristics and shocks were more likely to be associated with food insecurity [44, 45]. The regression modelling was specifically performed to determine the relationship between household food security and shocks experienced by households and to identify a combination of determinants of urban household food security. GLMM was used given the hierarchical nature of the data where individual households were nested onto the different administrative levels within the city. The data therefore violates the assumption of independence of respondents in standard logistic regression which could increase the chances of bias in the standard errors and hence the estimation of population parameters.

The ordinal cumulative logit link was used given the ordered nature of the dependent variable HFIAP. A stepwise analysis was adopted where the variables were entered into the model in four steps based on the three types of shocks experienced by households: economic, socio-political, or biophysical, while controlling for household socio-demographic characteristics. Table 2 presents the odds ratios for the independent variables accompanied with the 95% confidence interval and the associated p-values. Results are presented using adjusted odds ratio (OR) and 95% CI. The significance level of the findings is set at p-value < = 5%.

**Table 2:** Nairobi household demographic and economic characteristics.

		No.	%
Individual			
Sex of household head	Male	1,026	82.5
	Female	217	17.5
Age of household head	< = 24 years	105	8.4
	25–34 years	420	33.6
	35–44 years	392	31.4
	45–54 years	177	14.2
	55–64 years	69	5.5
	> = 65 years	87	7.0
	Extended	108	7.8
	Other	42	3.0
Place of birth of household head	Nairobi	266	21.3
	Another urban centre in Kenya	68	5.4
	Rural area in Kenya	874	70.0
	Foreign country	35	2.8
Duration of stay of household head in Nairobi	<5 years	69	7.9
	5–10 years	183	21.0
	>10 years	618	71.1
Household			
Type of household structure	Female-centred	239	17.3
	Male-centred	273	19.8
	Nuclear	752	54.5
	Extended	108	7.8
	Other	42	3.0
Main Household Income Source	Formal work	653	46.4
	Informal work	227	16.0
	Casual work	154	10.9
	Formal business	165	11.7
	Informal business	142	10.0
	Do not know/no response	70	5.0
Monthly Household Income	< = KSh10000.00	195	23.5
	KSh10001.00-KSh19000.00	140	16.8
	KSh19001.00-KSh34000.00	164	19.7
	KSh34001.00-KSh75000.00	166	20.0
	KSh75001.00+	166	20.0
Health Status of Household	No health issues	1156	81.8
	Some health issues	257	18.2

The analytical sample has a number of features that could compromise representativeness. First, the number of households surveyed in each sub-location was not strictly proportional to size at the time of the survey as the data on household numbers was only available for the 2009 Census. Second, various external factors impacted on the actual number of surveyed households in each sub-location. These included security considerations, the degree of cooperation from administrative officials, the availability of respondents during working hours, the willingness of potential respondents to participate and the limited access to gated communities. Third, because food insecurity is a long-term and often chronic condition, the study does not posit a causal relationship with the shocks experienced in the months prior to the survey. The presentation of results rather focuses on whether food insecurity increases the likelihood of vulnerability to shocks. Fourth, the data are based on participants’ self-reporting of shocks and household food security status and also assumes that household heads had accurate knowledge of all facets of their household characteristics, experience of shocks, and food security status. Finally, the data were collected two years prior to the COVID-19 pandemic and it is therefore not possible to draw any definitive conclusions about the actual impact of pandemic shocks. However, recent rapid-response surveys conducted during the pandemic do suggest that the impact has not been uniform across the city and that poorer, food insecure households were disproportionately affected [17–19].

## Results

### Household characteristics

Table 2 presents the socio-demographic and economic characteristics of the sampled households and household heads. The majority of household were male (83%). The survey instrument classifies household structure into four basic types: female-centred (female-head without a male spouse or partner); male-centred (male-head without a female spouse or partner); nuclear (male or female head with spouse or partner and immediate blood relatives); and extended (male or female head with spouse or partner plus more distant relatives and non-relatives). As many as 55% of the households surveyed were nuclear, while 20% were male-centred, and 17% were female-centred. In other words, all female-headed households were also female-centred. The average household size was 3.5 persons (with std dev of 1.452). Two thirds of the household heads were of working age between 25 and 44 years. Only 8% of heads were under the age of 25 and 7% over the age of 65. Nearly 80% of the household heads were migrants born outside Nairobi, with as many as 70% having migrated from a rural area. At the same time, only 8% of the household heads were recent migrants to the city, having lived in Nairobi for less than 5 years. Most were well-established with as many as 71% (including the 21% born in the city) having resided there for more than 10 years.

In terms of income source, 46% of the households reported that formal employment was their main source of income, while 15% had informal employment and 11% had casual work as their main source. Nearly 12% and 10% of the households relied on formal and informal businesses, respectively, as their main source of income. The fact that over one third of the households had no formal sector income source is consistent with the high rates of formal unemployment in the city, especially in the informal settlements. Income quintiles show that nearly a quarter of all households surveyed had a net monthly income of less than KSh10,000 (USD92) and that nearly 60% had an income of KSh34,000 (USD312) or less. Only 20% had a net monthly income of KSh75,000 (USD680) or more. Finally, with regard to health status, a total of 18% of the household members had a diagnosed medical condition with hypertension, asthma and diabetes most common.

### Household food insecurity

Table 3 shows that only 29% of households in the sample were completely food secure on the HFIAP scale at the time of the survey. All of the other households experienced some degree of food insecurity, including 36% who were mildly or moderately food insecure and 25% who were severely food insecure. Cross-tabulating the HFIAP by household type shows that extended, nuclear and male-centred households all had similar proportions of food secure and food insecure households. The major anomaly was female-centred households which were much more vulnerable to food insecurity. As few as 14% of female-centred households were food secure and as many as 44% were severely food insecure.

**Table 3:** Levels of household food insecurity in Nairobi by household structure.

	Total (N)		Female-Centred		Male-Centred		Nuclear		Extended		p-value
	No.	%	No.	%	No.	%	No.	%	No.	%	
Food Secure	410	29.2	65	14.3	73	26.7	216	28.9	39	36.5	0.137
Mildly Food Insecure	176	12.6	28	11.7	27	9.9	108	14.5	9	8.4	
Moderately Food Insecure	463	33.0	72	30.1	97	35.6	255	34.1	30	28.0	
Severely Food Insecure	353	25.2	74	43.9	76	27.8	168	22.5	29	27.1	
Total	1,402	100.0	239	100.0	273	19.8	747	100.0	107	100.0	

**Fig 1:**
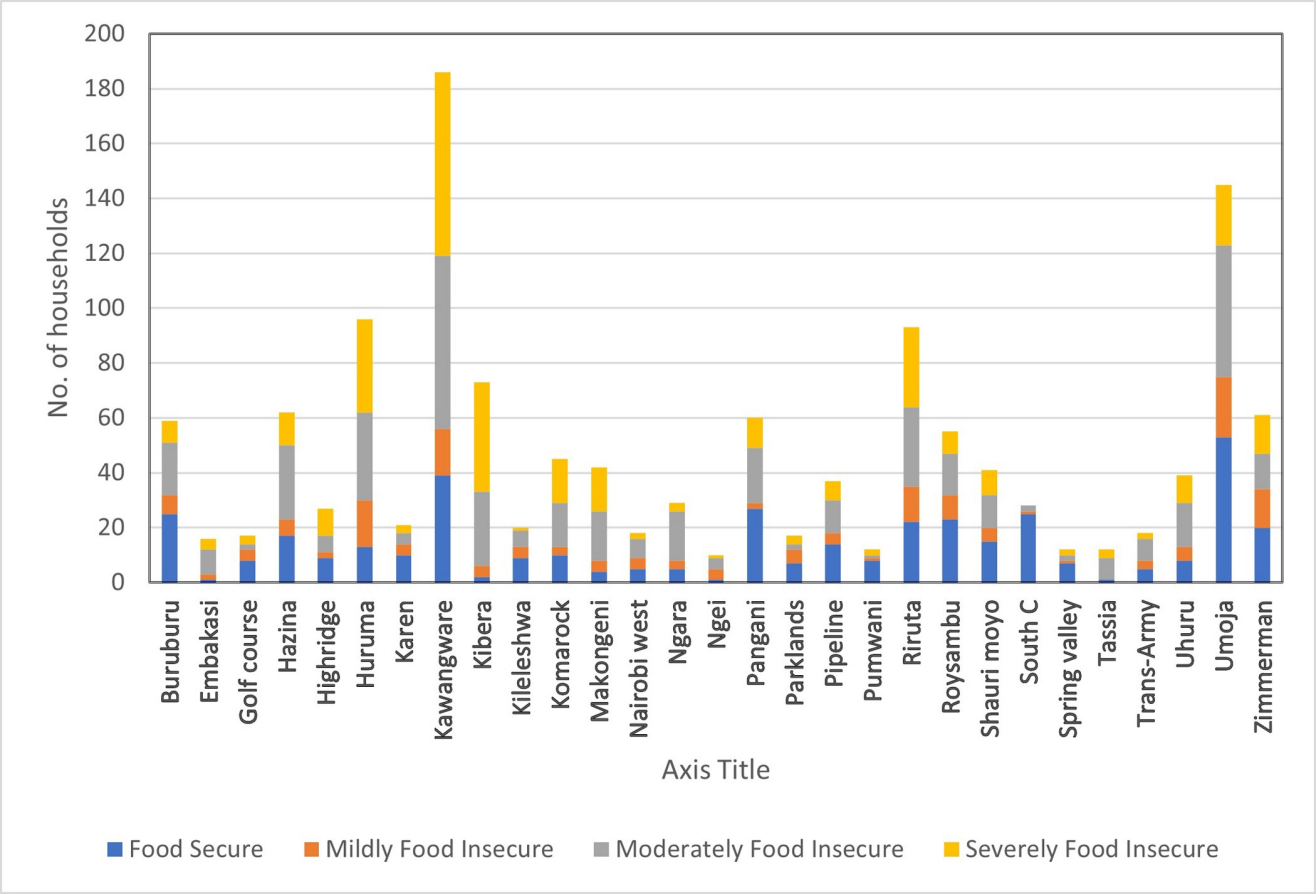
Household food security by Nairobi sub-location.

Fig 1 shows that there was also considerable variability in levels of food security and insecurity across the city. All urban communities had at least some food insecure households except for South C. Communities with informal settlements—including Kibera, Huruma and Kawangware—had a higher proportion of severely food insecure households, a finding that confirms earlier observations by other researchers that found that households in informal settlements were extremely vulnerable to food insecurity [22, 31].

### Household shocks

Table 4 shows the prevalence of experience of 16 different types of household shock in the six months prior to the survey. Economic shocks had been experienced by many more households than socio-political or biophysical shocks. Just over 60% had experienced going without food due to food price increases and 48% experienced a decline in income from a household member. Nearly one quarter (24%) had a household member who had lost their job. Of the possible socio-political shocks, theft of food or money was the most common (experienced by 6%), followed by insecurity or violence (5%). None of the biophysical shocks had been experienced by more than 4% of households. Conducted prior to COVID-19, a similar study now would probably show a significant increase in several economic shocks (especially loss of income and employment) and the biophysical health risks/epidemics shock.

**Table 4:** Experience of household shocks.

	No. of Households	% of Total
		Households
**Economic Shocks**	**763**	**55.0**
1 Going without foods due to price increases	825	60.5
2 Death of a working household member	35	2.5
3 Serious illness of a household member	158	11.2
4 Loss of employment for a household member	335	23.7
5 Reduced income of a household member	537	38.0
6 Reduction or cut-off of remittances	19	1.3
**Sociopolitical Shocks**	**226**	**16.3**
1 Insecurity/violence	64	4.5
2 Theft of money/food	85	6.0
3 Relocation of the family	29	2.1
4 Took in orphans due to death of parents	10	0.7
5 Political problems/issues	20	1.4
**Biophysical shocks**	**136**	**9.8**
1 Health risks/epidemics	26	1.8
2 Environmental hazards	13	0.9
3 Increased costs of water	40	2.8
4 Food cannot be safely stored	44	3.1
5 Lack of refrigeration for food	46	3.3

Note: Multiple response question.

### Relationship between food insecurity and household shocks

The results of the multivariate analysis of the Generalized Linear Mixed Models are presented in Table 5. The table provides the adjusted odds ratios (OR) for the independent variables using 95% CI. Model 1 controls for the socio-demographic characteristics of the household heads and households as a whole and shows that the odds of being food insecure are highest for households whose main source of income is casual wages and lowest for households with a regular wage. Households with an informal sector income were half as likely to be food insecure as those reliant on casual wages but more likely to be food insecure than those with a regular wage. The odds of being food insecure also increased as household income decreased. Households in the lowest income quintile were 14 times more likely to be food insecure than those in the upper quintile. Those in the other three income quintiles were 8, 6.5 and 3 times more likely to be food insecure respectively than the highest income households.

**Table 5:** Generalized linear mixed model of shocks to urban household food security.

Dependent/Independent variables	Model 1	Model 2	Model 3	Model 4
	OR (95%CI) ^p-value^	OR (95%CI) ^p-value^	OR (95%CI) ^p-value^	OR (95%CI) ^p-value^
HH Main Income Source (Ref = Informal business)				
Formal	0.763 (0.418–1.392)	0.695 (0.375–1.288)	0.767 (0.446–1.320)	0.803 (0.461–1.397)
Informal	1.473 (0.745–2.913)	1.050 (0.519–2.128)	1.262 (0.650–2.448)	1.328 (0.675–2.614)
Casual wage (formal & informal)	2.404 (1.121–5.155) **	1.625 (0.739–3.571)	1.803 (0.822–3.955)	1.772 (0.795–3.947)
Formal business	1.072 (0.513–2.242)	0.886 (0.412–1.904)	0.950 (0.455–1.983)	0.908 (0.426–1.937)
Net HH Income Without Loans (Ref = 75001.00+)				
< = 10000.00	14.902(7.21–30.798) ****	5.422 (2.770–10.613) ****	5.674 (2.893–11.127) ****	5.171 (2.635–10.146) ****
10001.00–19000.00	8.096(4.026–16.278) ****	2.340 (1.209–4.529) ***	2.298 (1.172–4.506) ***	2.336 (1.186–4.601) ***
19001.00–34000.00	6.507(3.359–12.607) ****	2.950 (1.638–5.311) ****	2.954 (1.631–5.347) ****	2.761 (1.515–5.030) ****
34001.00–75000.00	3.165 (1.691–5.924) ****	1.743 (1.010–3.010) **	1.716 (0.986–2.990) *	1.686 (0.959–2.966) *
HH health status (Ref = Unhealthy)				
Healthy	0.656 (0.407–1.057)	0.654 (0.418–1.025)	0.665 (0.418–1.057)	0.723 (0.449–1.163)
HH structure				
Female centred	11.452 (1.948–67.337) ***	10.577 (0.297–377.046)	8.768 (0.241–318.358)	8.134 (0.244–271.110)
Male centred	7.774 (1.517–39.841) ***	4.496 (0.141–143.419)	4.544 (0.14–147.527)	4.456 (0.147–135.331)
Nuclear	7.343 (1.445–37.320) ***	4.298 (0.137–134.551)	4.343 (0.136–138.475)	4.147 (0.140–123.147)
Extended	6.206 (1.075–35.819) **	3.931 (0.113–136.986)	4.222 (0.119–149.408)	3.617 (0.109–119.607)
Duration of stay in Nairobi (Ref = >10 years)				
<5 years	1.048 (0.534–2.055)	1.242 (0.614–2.511)	0.890 (0.412–1.922)	0.863 (0.403–1.846)
5–10 years	0.897 (0.569–1.413)	1.035 (0.642–1.667)	1.042 (0.643–1.687)	0.994 (0.611–1.617)
HH head place of birth (Ref = Foreign born)				
Nairobi	2.096 (0.556–7.898)	2.067 (0.100–42.645)	2.853 (0.586–13.880)	2.887 (0.494–16.885)
Another urban centre in Kenya	2.395 (0.638–8.993)	1.790 (0.395–8.105)	1.182 (0.265–5.267)	1.193 (0.252–5.658)
Rural area in Kenya	2.053 (0.590–7.144)	1.491(0.362–6.143)	0.946 (0.224–3.992)	0.971 (0.216–4.358)
**Household Shocks**				
**Economic shocks**				
1. Food price change				
Never		0.013 (0.002–0.077) ****	0.018 (0.003–0.097) ****	0.031 (0.005–0.192) ****
About once a month		0.100 (0.017–0.576) ***	0.128 (0.024–0.694) ***	0.204 (0.035–1.178) *
About once a week		0.170 (0.029–0.996) **	0.226 (0.042–1.212) *	0.350 (0.061–2.008)
> Once a week but <every day of the week		0.263 (0.044–1.555)	0.327 (0.060–1.778)	0.518 (0.088–3.062)
2. Death of a working HH member		1.178 (0.303–4.584)	0.994 (0.252–3.923)	1.076 (0.276–4.185)
3. Serious illness of HH member		0.837 (0.518–1.351)	0.837 (0.51–1.371)	0.793 (0.474–1.324)
4. Loss of employment for HH member		2.597 (1.674–4.029) ****	2.412 (1.543–3.772) ****	2.234 (1.404–3.553) ***
5. Reduced income of a HH member		1.783 (1.206–2.636) ***	1.678 (1.132–2.489) ***	1.637 (1.097–2.442) **
6. Reduced/ cut-off of remittances		3.539 (1.284–9.760) **	3.633 (1.337–9.873) ****	3.77 (1.278–11.163) ****
**Socio-political hazards**				
7. Insecurity/violence			1.877 (0.877–4.015)	1.789 (0.822–3.893)
8. Theft of money/food			0.946 (0.454–1.970)	0.968 (0.466–2.013)
9. Relocation of the family			2.505 (0.643–9.753) *	2.516 (0.609–10.384) *
10. Taking in orphans			1.526 (0.203–11.503)	1.638 (0.135–19.865)
11. Political problems/issues			1.032 (0.289–3.688)	1.126 (0.328–3.868)
**Biophysical hazards**				
12. Health risks/epidemics				1.792 (0.580–5.539)
13. Environmental hazards				3.520 (0.784–11.876)
14. Increased cost of water				1.163 (0.598–2.663)
15. Food cannot be safely stored				0.663 (0.286–1.534)
16. Lack of refrigeration for food				1.133 (0.522–2.460)

Legend: HH–Household; Net monthly income in Kenyan Shillings.

Significance level:

**** P≤ 0.001.;

*** P≤ 0.01.;

**P≤ 0.05.;

*P≤ 0.1.

Another finding in Model 1 is that type of household impacts the odds of being food insecure. Of the four main household types, female-centred households (11.452, 95% CI [1.948–67.337]) have the greatest chance of being food insecure and extended households the least. Male-centred (7.774, 95% CI [1.517–39.841]) and nuclear (7.343, 95% CI [1.445–37.320]) households have roughly the same odds of being food insecure, well below those of female-centred households. If everyone in the household is healthy (0.656, 95% CI [0.407–1.057]), the odds of being food secure increase.

Contrary to expectations that those who migrate to the city are likely to be more food insecure than those who were born there, Model 1 also shows that the chances of being insecure are similar if the household head was born in Nairobi, born in other Kenyan towns, or born in rural areas in Kenya. All three are twice as likely to be food insecure than households with foreign-born heads. The length of time a migrant household head has lived in Nairobi does not appear to significantly affect the odds of their household being food insecure.

Model 2 controls for the six different types of economic shock to the household. The relationships between the socio-demographic and economic characteristics of the household and food insecurity in Model 1 remained robust. In Model 2, the frequency of experiencing food price shocks emerged as the most significant driver of urban food insecurity, with the odds of being food insecure decreasing the less frequently the household experienced the impact. Households which had never experienced food price shocks (0.013, 95%CI [0.002–0.077]) were more likely to be food secure than those who had, even if that experience was only monthly or weekly. The Model also shows that loss of employment by a household member (2.597, 95% CI [1.674–4.029]) and a reduction in income (1.783, 95% CI [1.206–2.636]) also increased the odds of being food insecure when compared with households that experienced neither shock. While the cutting off of remittances (3.539, 95% CI [1.284–9.760]) was also associated with increased household food insecurity, less than 2% of households had experienced this shock.

Model 3 controls for six socio-political shocks including insecurity/violence, theft, death of or accident to a household member, relocation of the family, taking in of orphans, and political problems. The relationships identified in Models 1 and 2 remain robust in Model 3. The shock associated with increased odds of being food insecure included insecurity/violence and relocation (2.505, 95% CI [0.643–9.753]). Households that take in orphans (1.526, 95% CI [0.203–11.503]) were also more likely to be food insecure, but the numbers of households involved were small.

Model 4 included the biophysical shocks and after controlling for these, the other relationships remain robust. Households that experienced environmental hazards (3.52, 95% CI [0.784–11.876]) were more likely to be food insecure than those that did not. Similarly, Households that had experienced health/epidemic shocks (1.792, 95% CI [0.580–5.539]) showed an elevated risk of being food insecure.

In summary, at least three in every four households reported experiencing some level of food insecurity, with one in four reporting severe food insecurity. Female-centred households were almost twice as likely to be food insecure as other types of households. The socio-demographic characteristics of households including household income source, monthly income and household structure were also associated with household food security. Additionally, the odds of being food insecure increased as household income declined and that households with a formal wage income were more likely than households without a member in regular wage employment to be food secure. The modelling also showed that a household had a greater chance of being food secure if the household members were free of serious diagnosed medical conditions.

With regards to the various types of shocks to households, nearly six in ten households had experienced one or more of the economic shocks, and two in every ten and one in every ten experienced one or more of the sociopolitical and biophysical shocks respectively. Food price increases characterized by hikes in the cost of basic commodities are an important predictor of urban household food security status. The findings also show that the frequency of experiencing food price hikes increases the odds of a household being food insecure. Loss of employment and reduced income also significantly increased the odds of a household being food insecure. Of the various sociopolitical shocks, insecurity/violence and being forced to relocate the place of residence were most strongly associated with increased odds of being food insecure. Of the biophysical shocks, environmental hazards and health risks/pandemics are most strongly associated with food insecurity.

## Discussion

This study set out to investigate the association between household shocks and household food security in Nairobi. The results confirm that households that have experienced some form of socio-economic shock such as loss of employment, relocation, and reduced income have the highest chance of being food secure. Also, rapid and frequent change in the price of basic food commodities increases the odds of being food insecure. While the production of food and its availability in the market are important dimensions of food security, the analysis confirms that income and economic access to the available food are more important to urban populations. This combined with the effects of overcrowding, an unhygienic environment, and the weak social grant system makes many households, especially in the informal settlements, extremely vulnerable to future shocks and food insecurity.

Food security in Kenya has traditionally been omitted by city planners and managers in urban development plans despite its centrality to the health and wellbeing of any human population. There are several reasons for this. First, food insecurity has been seen as an essentially rural and agricultural production rather than urban and food access challenge. However, food insecurity in cities is not necessarily linked to seasonal agricultural changes or other community-wide phenomena, as in rural areas, but is rather a function of individual and household fortunes in the labour market and the informal economy. One of the consequences of the lack of integration of food security into development planning is that emergency food preparedness planning has not been viewed as a priority. Rather, emergency procedures are only enacted when a food emergency is already in progress. Second, there is an implicit assumption that creating employment and improving urban infrastructure will guarantee urban food security. While there is growing evidence in other contexts that both strategies do produce better food security outcomes, in Nairobi it means that a better understanding of the urban food system and the specific drivers of and remedies for food insecurity need to be further explored. Finally, as in many other African cities, Nairobi’s food system planners have placed undue emphasis on urban agriculture as a panacea for food insecurity in cities in the past, to the neglect of other mitigators of food insecurity at times of acute distress. This was particularly evident in the passage of the 2015 Urban Agriculture Promotion and Regulation Act [46].

The fourth draft of Nairobi’s new food system strategy “acknowledges that Nairobi City food system is presently not able to deliver adequate amounts of safe, nutritious and good quality food to all the city residents nor afford good benefits” [47]. The strategy has four main objectives: increased food production in Nairobi and rural counties supplying food to the city, stability of food supply and incomes, reduction of food losses, and the good welfare of food consumers. The latest draft proposes that the City will institute a contingency plan with early warning and action components including (a) early warning food and nutrition assessment in the community; (b) stockpiling of food to be distributed in emergencies, with stockpiling to be contracted to the private sector; (c) registration of vulnerable persons together with public education and community outreach in the “alert phase”; (d) mapping of vulnerable persons during the “alarm” phase; and (e) food relief and other social protection measures during the “emergency” phase [47]. In 2017, Nairobi also started to implement an Urban Early Warning and Early Action (UEWEA) Initiative on food security in partnership with Concern Worldwide, Kenya Red Cross, and Oxfam [48]. Amongst its aims are the set-up of a coordinated urban early action mechanism within the city; strengthening the capacity of six Nairobi sub-counties and one informal settlement community to mitigate and respond quickly to the impacts of slow onset emergencies; and ensuring routine surveillance in urban informal settlements.

These new food system governance initiatives around preparedness present an important opportunity for evidence-based interventions in Nairobi that are not simply activated in the midst of an emergency. The approach adopted in this paper adds value to these policy initiatives by (a) identifying which types of households across the city are most vulnerable to food insecurity, and (b) analyzing the relationship between household food security and a range of economic, biophysical, and socio-political shocks experienced by those households. The paper demonstrates that in the months leading up to the survey, households were more likely to experience economic shocks (food price increases, loss of employment and reduced income in particular) than sociopolitical or biophysical shocks. Of course, we cannot conclude this is always the case as periodic political conflict and terrorist attacks have affected Nairobi in recent years [49, 50]. Also, the COVID-19 pandemic would qualify as a biophysical shock in our classification to the extent that it impacted on the health of household members. However, its primary shock at the household level has been economic in nature.

## Conclusion

The vulnerability of households to economic and other potential shocks have been starkly exposed by the COVID-19 pandemic which is having a major impact on levels of food insecurity in Nairobi, primarily through reduced employment and livelihood opportunities for the urban poor. Food prices have also surged in the wake of the pandemic [21]. As this paper has demonstrated, vulnerability to shocks is not a new phenomenon in Nairobi and even prior to the pandemic policy-makers in the city had begun to turn their attention to the need for emergency preparedness strategies. The fourth draft of the Nairobi City Country Food System Strategy, issued in November 2020 makes no reference to the challenges of COVID-19 but it does suggest the rudiments of a future preparedness strategy focused on the most vulnerable households. While the Strategy suggests that the identification of vulnerability should be part of a reactive strategy, this paper’s findings indicate that it is necessary to be more proactive and identify types of vulnerable households most likely to be affected by sudden shocks, pandemic-related and otherwise. In particular, female-centred households, households without a wage income, and low-income households are all particularly vulnerable to food insecurity and to economic, sociopolitical, and biophysical shocks. Mapping of these households does not have to wait until a shock is upon the city hut could profitably and proactively be pursued as part of the finalized food system strategy for the city.

## References

[1] Ansah I, Gardebroek C, Ihle R Resilience and household food security: a review of concepts, methodological approaches and empirical evidence. Food Secur. 2019; 11:1187–11203.

[2] Akter S, Basher S. The impacts of food price and income shocks on household food security and economic well-being: Evidence from rural Bangladesh. Glob Environ Change. 2014; 25:150–162.

[3] Heltberg R, Oviedo A, Talukdar F. What do household surveys really tell us about risk, shocks, and risk management in the developing world? J Dev Stud. 2015; 51:209–225.

[4] Lokonon B, Savadogo K, Mbaye A. Assessing the impacts of climate shocks on farm performance and adaptation responses in the Niger Basin of Benin. Af J Agric Res Econ. 2015; 10:234–249.

[5] Misselhorn A. What drives food insecurity in southern Africa? A meta-analysis of household economy studies. Glob Environ Change. 2005; 15:33–43.

[6] Niles M, Salemo J. A cross-country analysis of climate shocks and smallholder food insecurity. PLOS ONE 2018; 13(2):e0192928. doi: 10.1371/journal.pone.0192928 29474383 PMC5825057

[7] Crush J, Riley L. Rural bias and urban food security. In: Battersby J, Watson V, editors. Urban food system governance and poverty in African cities London: Routledge; 2019. pp. 42–55.

[8] Frayne B. Pathways of food: mobility and food transfers in southern African cities. Int Dev Plan Rev. 2010; 32:291–310.

[9] Owuor S. The State of Household Food Security in Nairobi, Kenya. Cape Town, and Waterloo.: HCP Report No. 11; Cape Town, South Africa and Waterloo, Canada. 2018.

[10] Aday S, Aday M. Impact of COVID-19 on the food supply chain. Food Qual Safety. 2020; 4:167–180. 10.1093/fqsafe/fyaa024 PMC7499675 40504224

[11] Galanikis C. The food systems in the era of the coronavirus (COVID-19) pandemic crisis. Foods. 2020; 5:23. 10.3390/foods9040523 PMC7230343 32331259

[12] Laborde D, Martin W, Swinnen J, Vos R. COVID-19 risks to global food security. Science. 2020; 369:500–502. doi: 10.1126/science.abc4765 32732407

[13] Shilomboleni H. COVID-19 and food security in Africa: Building more resilient food systems AAS Op Res. 2020; 3(27). doi: 10.12688/aasopenres.13078.1 32734141 PMC7376614

[14] Devereux S, Béné S, Hoddidinott J. Conceptualising COVID-19’s impacts on household food security. Food Secur. 2020; 12:769–772. doi: 10.1007/s12571-020-01085-0 32837651 PMC7358330

[15] Iheme G, Jagun A, Egechizuorom I, Ogbonna O, Edafioghor L, Adeleke F, et al. Food consumption and coping strategies of urban households in Nigeria during the COVID-19 pandemic lockdown. World Nutr. 2020; 11(3):35–50

[16] Ouko K, Gwada G, Alworah Z, Onganga S, Ochieng J. Effects of Covid-19 pandemic on food security and household livelihoods in Kenya. Rev Ag App Econ. 2020; 23:72–80.

[17] Kansiime M, Tambo J, Mugambi I, Bundi M, Kara A, Owuor C. COVID-19 implications on household income and food security in Kenya and Uganda: findings from a rapid assessment. World Development. 2021; 137:105199. doi: 10.1016/j.worlddev.2020.105199 32982018 PMC7500897

[18] Nyadera I, Onditi F. COVID-19 experience among slum dwellers in Nairobi: A double tragedy or useful lesson for public health reforms? Int Soc Work. 2020; 63:838–841.

[19] Nechifor V, Ramos M, Ferrari E, Laichena J, Kihiu E, Omanyo D, et al. Food security and welfare changes under COVID-19 in sub-Saharan Africa: Impacts and responses in Kenya. Glob Food Secur. 2021; 28:100514. doi: 10.1016/j.gfs.2021.100514 33738191 PMC7938699

[20] KNBS. Survey on socio economic impact of COVID-19 on households report, wave two. Nairobi: National Bureau of Statistics; 2020.

[21] Kiria Chege C., Mbugua, M., Onyango, K., and Lundy, M. Keeping food on the table: Urban food environments in Nairobi under COVID-19. CIAT Publication No. 514. International Center for Tropical Agriculture (CIAT). Nairobi, Kenya; 2021.

[22] Kimai-Murage E, Schofield L, Wekesah F, Mohamed S, Mberu B, Ettarh R, et al. Vulnerability to food insecurity in urban slums: experiences from Nairobi, Kenya. J Urb Health. 2014; 91:1098–1113. 10.1007/s11524-014-9894-3 PMC4242851 25172616

[23] Ansah I, Gardebroek C, Ihle R. Shock interactions, coping strategy choices and household food security. Clim Dev. 2021; 13:414–426.

[24] Haysom., and Fuseni, I. Urban Social Protection and Food Systems: Lessons from South Africa In: Frayne B, Crush J, McCordic C, editors. Food and Nutrition Security in Southern African Cities New York: Routledge: Routledge; 2018. p. 66–85.

[25] Tevera D., and Simelane, N. Urban Food Insecurity and Social Protection. In: Crush J, Battersby, J., editor. Rapid Urbanisation, Urban Food Deserts and Food Security in Africa. Cham: Springer; 2016. p. 157–68.

[26] Verpoorten J, Arora A, Stoop N, Swinnen J. Self-reported food insecurity in Africa during the food price crisis. Food Pol. 2013; 39:51–63.

[27] Tongruksawattana S, Wainaina P. Climate shock adaptation for Kenyan maize-legume farmers: Choice, complementarities and substitutions between strategies. Clim Dev. 2019; 11:710–722.

[28] FAO. Food security policy brief. Rome: Food and Agriculture Organization (FAO); 2006.

[29] Owuor S. Urbanization and household food security in Nairobi, Kenya. In: Nagao M, Masinja J, Alhassan A, editors. Sustainable development in Africa: Case studies. Denver, USA: Spears Media Press; 2019. pp. 161–74.

[30] Ahmed S, Haklay M, Tacoli C, Githiri G, Dávila J, Allen A, et al. Participatory mapping and food-centred justice in informal settlements in Nairobi, Kenya. Geo: Geog Environ. 2019; 6.

[31] Mohamed S, Mberu B, Amendah D, Kimani-Murange E, Ettarh R, Schofield L, et al. Poverty and uneven food security in urban slums In: Crush J, Battersby J, editors. Rapid urbanisation, urban food deserts and food security in Africa. Cham: Springer; 2016. pp. 97–112.

[32] Wager J, Hinton L, McCordic C, Owuor S, Capron G, Arreleano S. Do urban food deserts exist in the global South? An analysis of Nairobi and Mexico City. Sustainability. 2019; 11(7): 1963.

[33] Schofield L, Mohamed S, Kimani-Murage E., Wekesah F, Mberu B, Egondi T, et al. Spotting the invisible crisis: Early warning indicators in urban slums of Nairobi Kenya. ENNONLIN, 2013. Available from: https://www.ennonline.net/fex/46/spotting.

[34] Corburn J, Vlahov D, Mberu B, Riley L, Caiaffa W, Rashid S, et al. Slum health: Arresting COVID-19 and improving well-being in urban informal settlements J Urban Health. 2020; 97:348–357. doi: 10.1007/s11524-020-00438-6 32333243 PMC7182092

[35] Hamilton H, Henry R, Rounsevell M, Moran D, Cossar F, Allen K, et al. Exploring global food system shocks, scenarios and outcomes. Futures. 2020; 123:102601. doi: 10.1016/j.futures.2020.102601 32836328 PMC7320689

[36] Wien M, Sabate J. Food selection criteria for disaster response planning in urban societies. Nutr J. 2015; 14(47). doi: 10.1186/s12937-015-0033-0 25962636 PMC4489367

[37] Farrell P., Thow A, Wate J, Nonga N, Vatucawaqa P, Brewer T, et al. COVID-19 and Pacific food system resilience: Opportunities to build a robust response Food Secur. 2020; 12: 783–791. 10.1007/s12571-020-01087-y PMC7369468 32837656

[38] Darma S, Pusriadi T, Syaharuddin S, Darma D. Indonesia government’s strategy for food security during the COVID-19 period 2020. Int J of Adv Sci Tech. 2020; 29(4):10338–10348.

[39] Cardwell R, Ghazalian P. COVID-19 and international food assistance: Policy proposals to keep food flowing. World Dev. 2020; 135: 105059. doi: 10.1016/j.worlddev.2020.105059 32834375 PMC7321024

[40] Zhong T, Si Z, Crush J, Scott S. COVID-19 and emergency food security policies in Wuhan and Nanjing China. Dev Pol Rev (forthcoming). doi: 10.1111/dpr.12575 34548764 PMC8444884

[41] High Level Panel of Experts. Impacts of COVID-19 on food security and nutrition: developing effective policy responses to address the hunger and malnutrition pandemic. Rome: World Committee on Food Security; 2020: 34.

[42] Coates J, Swindale A, Bilinsky P. Household Food Insecurity Access Scale (HFIAS) for Measurement of Food Access. Washington DC: FANTA; 2007.

[43] Coates J. Build it back better: Constructing food security for improved measurement and action. Glob Food Sec. 2013; 2:188–94.

[44] Schielzeth H, Nakagawa S. Nested by design: Model fitting and interpretation in a mixed model era. Methods Ecol Evol. 2013; 4:14–24.

[45] Skrondal A, Rabe-Hesketh S. Some applications of Generalized Linear Latent and Mixed Models in epidemiology: Repeated measures, measurement error and multilevel modeling. Nor Epidemiol. 2003; 13.

[46] Republic of Kenya. Urban Agriculture Promotion and Regulation Act, 2015. Nairobi: Nairobi City Council; 2016.

[47] Nairobi City Council. Nairobi City Country Food System Strategy: Fourth Draft. Nairobi: Nairobi City Council, FAO and C40 Cities; 2020.

[48] START Network. Urban Early Warning Early Action (UEWEA) Project in Kenya: Food and Nutrition (Nairobi). 2017.

[49] Mueller J. The evolution of political violence: The case of Somalia’s Al-Shabaab. Terr Pol Viol. 2018; 30:116–41.

[50] Mueller S. Dying to win: Elections, political violence, and institutional decay in Kenya J Cont Af Stud 2011; 29:99–117.

